# Changing Dental Profession—Modern Forms and Challenges in Dental Practice

**DOI:** 10.3390/ijerph18041945

**Published:** 2021-02-17

**Authors:** Thomas Gerhard Wolf, Guglielmo Campus

**Affiliations:** 1 Department of Restorative, Preventive and Pediatric Dentistry, School of Dental Medicine, University of Bern, CH-3010 Bern, Switzerland; guglielmo.campus@zmk.unibe.ch; 2 Department of Periodontology and Operative Dentistry, University Medical Center of the Johannes Gutenberg-University Mainz, 55131 Mainz, Germany

**Keywords:** artificial intelligence, big data, challenges, changing dental profession, COVID-19, cross-border use of services, dental practice, dental personnel, dentist, digitalization, expectations, e-health, future career prospects, green dentistry, health care, modern forms of practice, one health approach, oral health policy, pandemic, SARS-CoV-2, sustainability, tele-dentistry, worldwide, young dentists

In the last two decades, an increasing trend towards new forms of dental practice was observed. The classic model of a dental practice with only one dentist is still the most common form of practice, but requirements and political conditions have largely changed and continue to change [1]. Nowadays, young dentists have several job opportunities, i.e., being a self-employed intrapreneur or being an employee in one of the practice forms, or working in the public health sector or in industry. Career prospects are diverse and much less clear-cut than in the past.

The oral healthcare sector is shifting due to factors like financial investors, with the opening of many large dental centers or even chains, an increasing number of employed dentists, a possible oversupply in urban and undersupply in rural areas and the shortage or the migration of skilled personnel. Furthermore, technical and professional challenges associated with progress through research [2], new fields such as artificial intelligence and big data, sustainability and green dentistry, tele-dentistry, the cross-border use of services and the digital transformation in health care contribute to change. These factors can influence dentists, people working in the oral healthcare sector and even patients. A reorganization of public and private dentalcare providers’ activities might be required [3].

This necessarily includes the potential consequences and impact of the SARS-CoV-2/COVID-19 pandemic [4,5], the end of which, although not currently foreseeable worldwide, will nevertheless have an immense impact on the dental practice landscape. These include, for example, changed practice procedures due to changed or extended hygiene standards, a reduction in jobs at one location and a need for new skills and jobs such as telemedicine applications at another location, changed patient flows and modified therapy options due to a possible loss of health insurance or improved training of staff to deal with pandemic situations. Consequences must also be discussed, such as effects of the pandemic on dental personnel such as anxiety or sleep disturbances.

All the challenges and possible influences that are changing the dental profession are welcomed to the current Special Issue entitled “Changing Dental Profession–Modern Forms and Challenges in Dental Practice” to analyze, develop solutions and support for dental personnel and for creating and forming the future dental practice.

## Data Availability

Not applicable.
